# Supplementary material for: HIV Increases the Inhibitory Impact of Morphine and Antiretrovirals on Autophagy in Primary Human Macrophages: Contributions to Neuropathogenesis
Source: Cells. 2021 Aug 24;10(9):2183. doi: 10.3390/cells10092183 (PMC8470112; doi:10.3390/cells10092183)
Supplement: Supplementary file 1 [file cells-10-02183-s001.zip › cells-1281583-supplementary.pdf]

Supplemental Files

Supplementary Figures

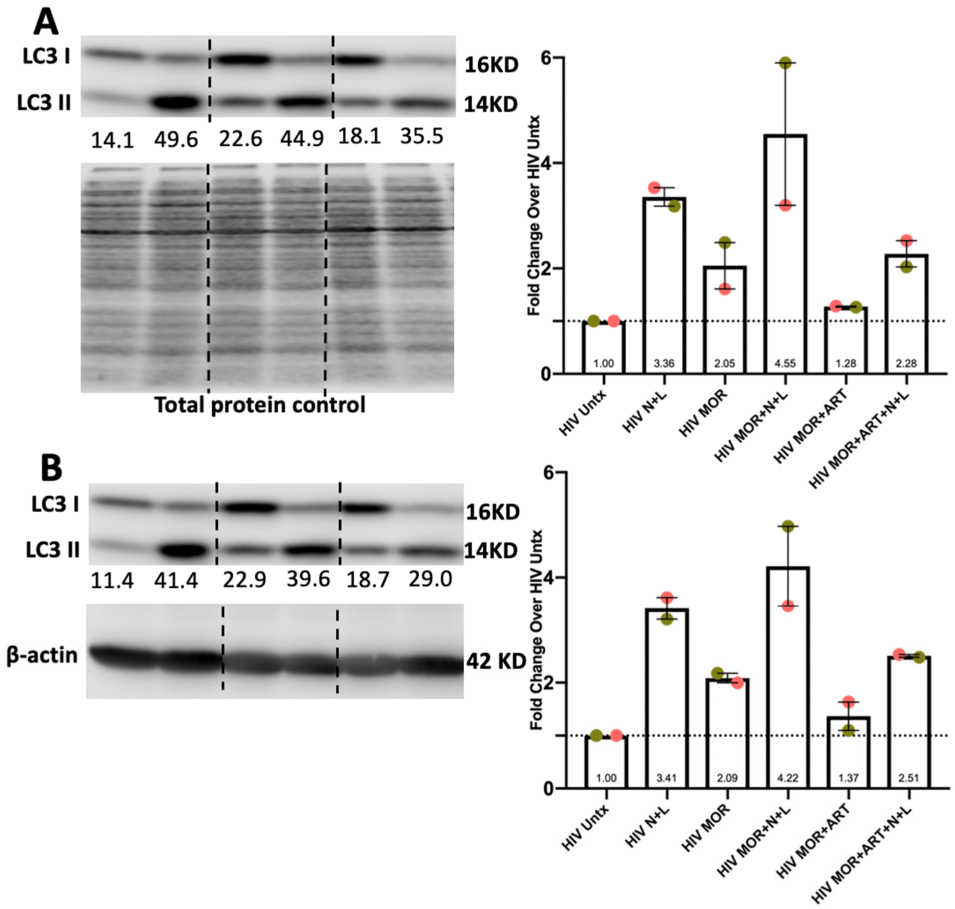

Supplementary Figure 1. Western blotting comparison of total protein loading control compared to β-actin loading control for two experiments in HIV-infected MDM. Primary human macrophages were cultured from PBMC as described in the Materials and Methods section, infected with HIV for 3-4 days, and treated with morphine with/without ART for 24h. The numbers below the LC3II bands represent the raw LC3II values normalized to either the total protein or acting loading control presented below. **(A)** Sample Western blot with total protein loading control, LC3II values relative to total protein, and LC3II levels normalized to the untreated control set to 1.0 for two experiments. **(B)** Sample Western blot with β-actin loading control, LC3II values relative to β-actin, and LC3II levels normalized to the untreated control set to 1.0 for two experiments, n = 2 independent experiments and error bars represent SEM.

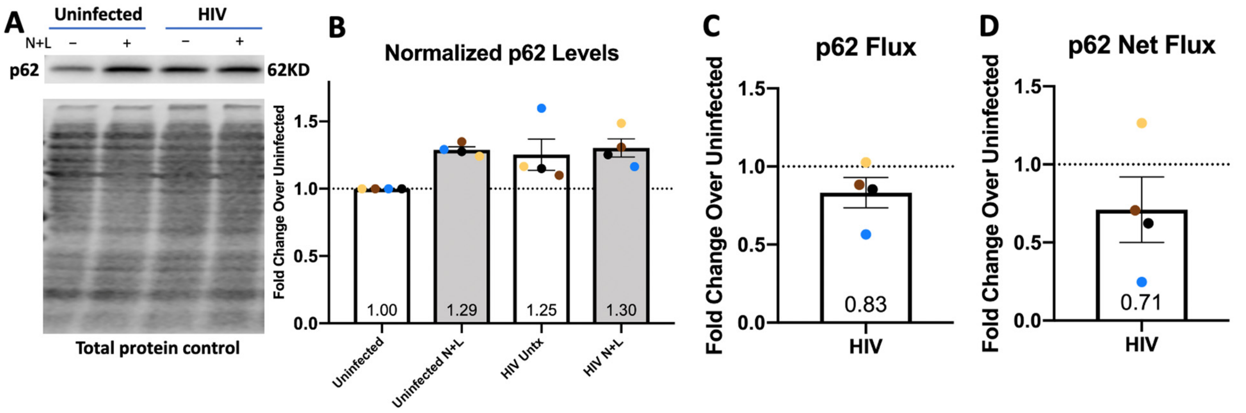

Supplementary Figure 2. Normalized p62 levels, p62 flux, and p62 net flux analyzed by Western blotting in primary human macrophages. **(A)** Representative Western blot for untreated uninfected MDM vs HIV-infected MDM with total protein loading control. **(B)** p62 protein levels relative to total protein and then normalized to the untreated control for each experiment. **(C)** p62 flux, equivalent to the rate of p62 degradation by autophagy per treatment condition relative to control set at the dotted line at 1.0, was quantified. **(D)** p62 net flux, equivalent to the amount of p62 degradation by autophagy per treatment condition relative to control set at the dotted line at 1.0, was quantified. Error bars depict SEM, $n = 4$ independent experiments.

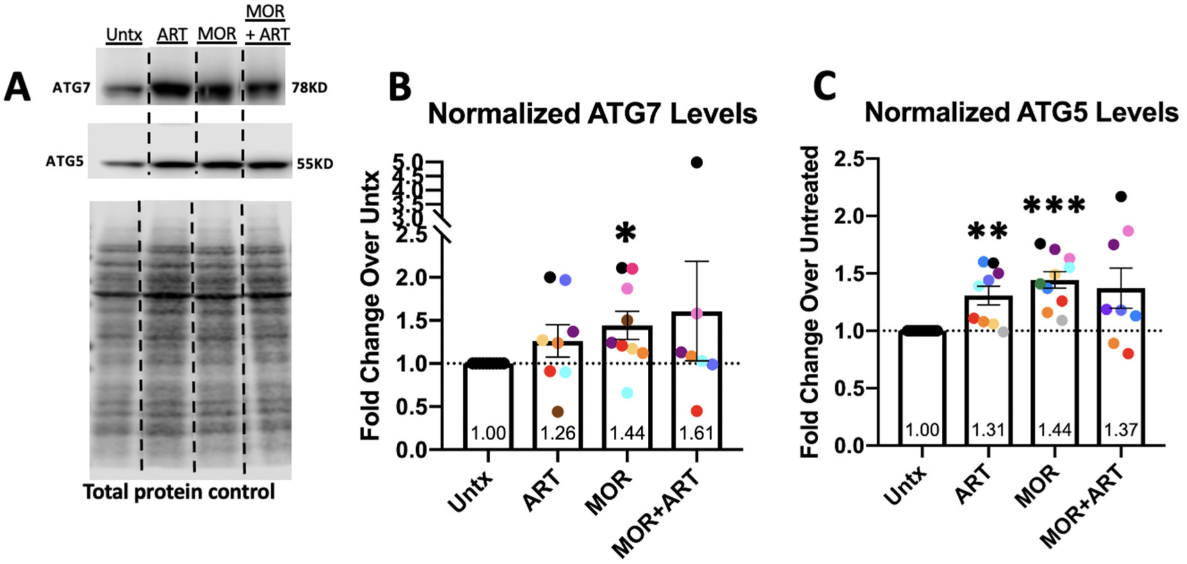

Supplementary Figure 3. Normalized ATG7 and ATG5 levels analyzed by Western blotting in primary human macrophages. **(A)** Representative Western blot for ATG7 (Cell Signaling Cat#8558 1:1000 dilution) and ATG5 (Cell Signaling Cat#12994 1:1500 dilution) in uninfected MDM treated with morphine and/or ART. **(B)** Normalized ATG7 levels relative to the untreated (Untx) control set to 1.0 at the dotted line. **(D)** Normalized ATG5 levels relative to control set to 1.0 at the dotted line. Error bars represent SEM, n = 7-10 independent experiments, *p<0.05 **p<0.01 ***p<0.001 one sample t-test.

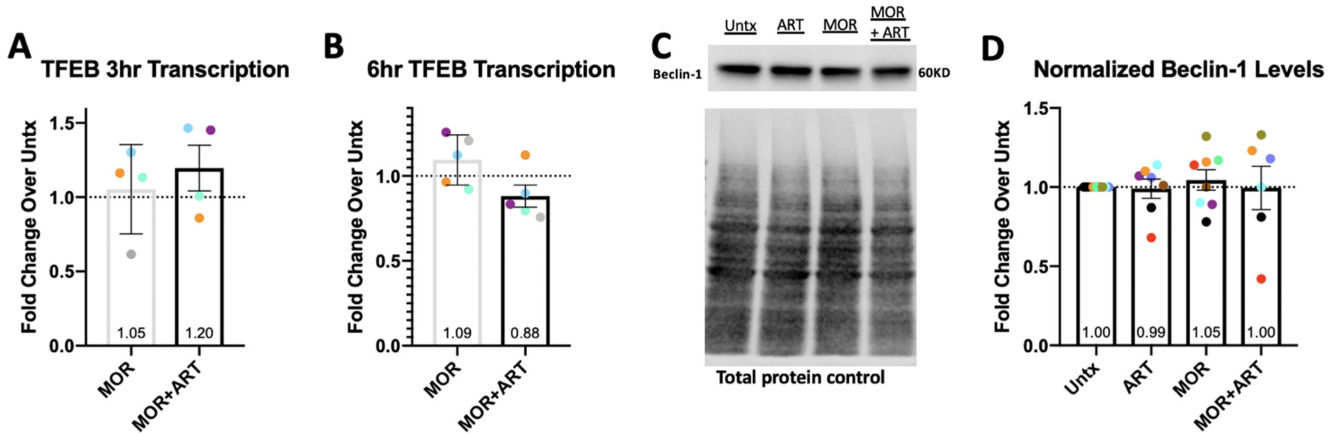

Supplementary Figure 4. *TFEB* mRNA transcription in uninfected MDM treated with morphine +/- ART and BECLIN-1 expression by Western blotting. **(A)** *TFEB* (Thermo Fisher Primer ID#Hs00292981_m1) transcription relative to the untreated (Untx) control set to 1.0 after 3hr treatment. **(B)** *TFEB* transcription relative to the untreated control set to 1.0 after 6hr treatment. **(C)** Representative Western blot for BECLIN-1 (Cell Signaling Technology Cat#4122 1:1000 dilution) in uninfected MDM treated with morphine and/or ART for 24h. **(D)** Normalized BECLIN-1 levels relative to the untreated control set to 1.0 at the dotted line. Error bars represent SEM, n = 4-8 independent experiments.

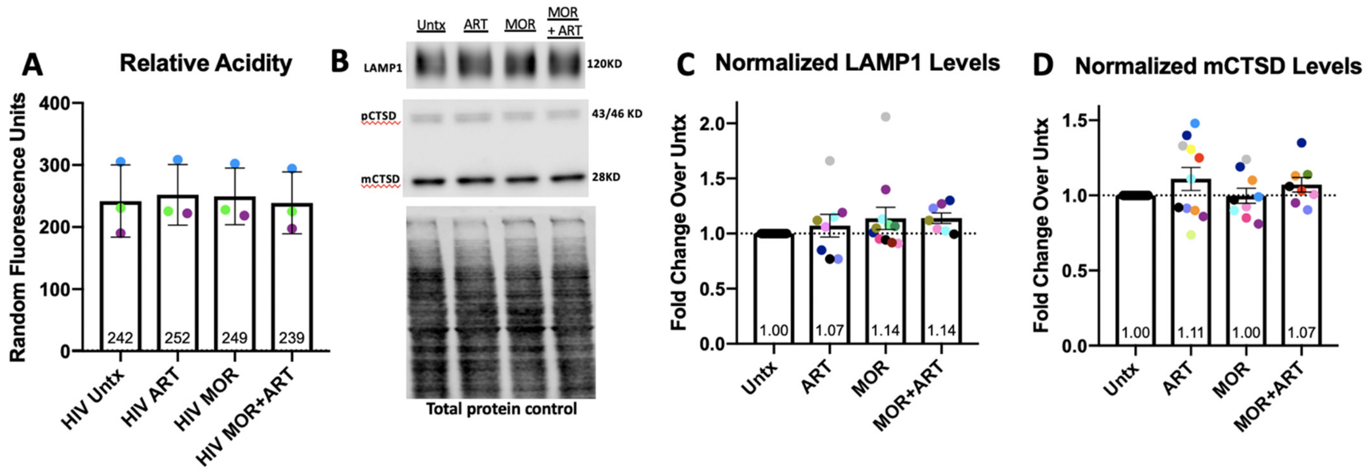

Supplementary Figure 5. Intracellular pH in HIV-infected MDM by a fluorometric plate reader assay and levels of LAMP1 and mature Cathepsin D (mCTSD) in uninfected MDM by Western blotting. **(A)** HIV-infected MDM were stained for 30 min with pHrodo™ Red AM Intracellular pH Indicator (Invitrogen Cat#P35372) according to the manufacturer's protocol. Dye-loaded cells were untreated (HIV Untx) or treated with morphine and/or ART for 24h, and relative acidity was quantified by a Spectra Max M5 fluorometer (Molecular Devices) with Ex: 560nm Em: 585nm. **(B)** Representative Western blot for LAMP1 (abcam Cat#ab24170 1:1500 dilution) and mCTSD (abcam Cat#ab75852 1:10000 dilution) in uninfected MDM treated with morphine and/or ART for 24h. **(C)** Normalized LAMP1 levels relative to the untreated (Untx) control set to 1.0 at the dotted line. **(D)** Normalized mCTSD levels relative to the untreated control set to 1.0 at the dotted line. Error bars for acidity represent SD and error bars for Western blotting results represent SEM, *n* = 3-11 independent experiments.

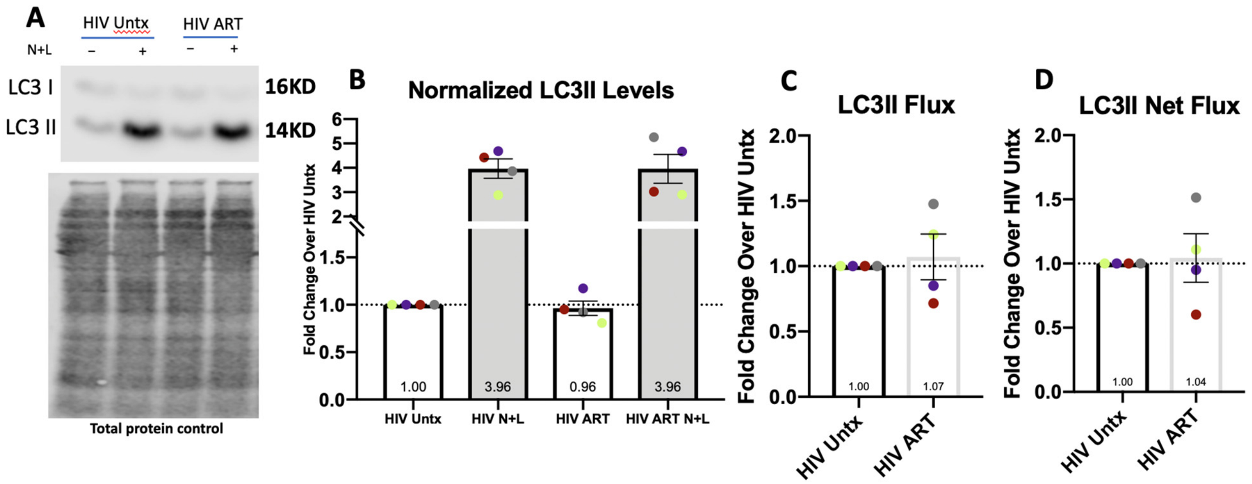

Supplementary Figure 6. Normalized LC3II levels, LC3II flux, and LC3II net flux analyzed by Western blotting in HIV-infected primary human macrophages treated

with ART for 24h. **(A)** Representative LC3II Western blot for untreated (HIV Untx) MDM and ART-treated MDM (HIV ART). **(B)** LC3II protein levels relative to total protein and then normalized to the untreated control for each experiment. **(C)** LC3II flux relative to control set to 1.0 at the dotted line. **(D)** LC3II net flux relative to control set to 1.0 at the dotted line. Error bars represent SEM, n = 4 independent experiments.
